# Wnt Signaling Pathway Dysregulation in the Aging Brain: Lessons From the *Octodon degus*

**DOI:** 10.3389/fcell.2020.00734

**Published:** 2020-08-05

**Authors:** Nibaldo C. Inestrosa, Cheril Tapia-Rojas, Carolina B. Lindsay, Juan Manuel Zolezzi

**Affiliations:** ^1^Centro de Envejecimiento y Regeneración (CARE), Departamento de Biología Celular y Molecular, Facultad de Ciencias Biológicas, Pontificia Universidad Católica de Chile, Santiago, Chile; ^2^Centro de Excelencia en Biomedicina de Magallanes (CEBIMA), Universidad de Magallanes, Punta Arenas, Chile

**Keywords:** Wnt signaling, aging, *O. degus*, neurodegeneration, Alzheimers’ disease

## Abstract

Wnt signaling constitutes a fundamental cellular and molecular pathway, necessary from proper embryogenesis to function-maintenance of fully developed complex organisms. In this regard, Wnt pathway plays a crucial role in both the development of the central nervous system and in maintaining the structure and function of the neuronal circuits, and it has been suggested that its dysregulation is critical in the onset of several pathologies including cancer and neurodegenerative disorders, such as Alzheimer’s disease (AD). Due to its relevance in the maintenance of the neuronal activity and its involvement in the outbreak of devastating diseases, we explored the age-related changes in the expression of Wnt key components in the cortex and hippocampus of 7 to 72-months-old *Octodon degus* (*O. degus*), a Chilean long-living endemic rodent that has been proposed and used as a natural model for AD. We found a down-regulation in the expression of different Wnt ligands (Wnt3a, Wnt7a, and Wnt5a), as well as in the Wnt co-receptor LRP6. We also observed an increase in the activity of GSK-3β related to the down-regulation of Wnt activity, a fact that was confirmed by a decreased expression of Wnt target genes. Relevantly, an important increase was found in secreted endogenous Wnt inhibitors, including the secreted-frizzled-related protein 1 and 2 (SFRP-1 and SFRP-2) and Dickkopf-1 (Dkk-1), all them antagonists at the cell surface. Furthermore, treatment with Andrographolide, a labdane diterpene obtained from *Andrographis paniculata*, prevents Wnt signaling loss in aging *degus*. Taken together, these results suggest that during the aging process Wnt signaling activity decreases in the brain of *O. degus*.

## Highlights

- The aging process involves the dysregulation of the Wnt signaling pathway, including ligands, downstream effectors and Wnt target genes in both hippocampus and cortex of *O. degus*.
- Soluble endogenous inhibitors of Wnt signaling pathway increase in an age-dependent manner in both hippocampus and cortex of *O. degus*.
- Age-related Wnt signaling impairments in *O. degus* were recovered by Andrographolide (ANDRO) treatment.

## Introduction

Increased aging of the world population has become a worldwide concern mainly because the close relationship between age and the appearance of different pathologies. Indeed, aging is considered the main risk factor for several pathologies, including cancer, and neurodegenerative disorders, such as Alzheimer’s disease (AD) and Parkinson’s disease, among others (Inestrosa and Toledo, 2008; Nusse and Clevers, 2017; Oliva et al., 2018; Steinhart and Angers, 2018; Palomer et al., 2019).

Behind the aged phenotype, a relevant feature of the aging process is the gradual loss of activity or alteration of several molecular components necessary for cell physiology. Molecular pathways, which usually encompass an amply range of biological molecules, drive the different cellular processes and, ultimately, determine the cellular fate. In this regard, the signaling pathways mediated by the Wnt ligands are involved in diverse aspects of cell-cell communication, including the regulation of cell proliferation, the occurrence of fibrosis, and cellular morphogenesis (Cisternas et al., 2014; Fuenzalida et al., 2016; Gammons and Bienz, 2018). Currently, 19 Wnt ligands have been described in vertebrates, which may initiate either of two signaling pathways called the canonical and the non-canonical pathways (Nusse and Clevers, 2017; Oliva et al., 2018). Relevantly, although Wnt pathway has been recognized as critical for the central nervous system development, several Wnt components retain their expression in the adult brain, including the hippocampus, and have proven to be fundamental in both the development and function of synapses (Inestrosa and Arenas, 2010; Inestrosa and Varela-Nallar, 2015). Indeed, different studies have indicated a strong correlation between Wnt signaling alteration and the appearance of neurodegenerative disorders, such as AD (Caricasole et al., 2004; Inestrosa and Toledo, 2008; Garcia-Velazquez and Arias, 2017). In this particular case, it is clear that the expression of some Wnt components change during the progression of AD, such as β-catenin which was reduced in patients carrying presenilin-1-inherited mutations (Zhang et al., 1998). Moreover, Wnt signaling activation can inhibit the formation of the amyloid-β peptide (Aβ) aggregates; and Apolipoprotein E ε4, the main risk factor for AD, can inhibits Wnt signaling (Roses, 1994; Liu M. et al., 2014). Altogether, these findings strongly suggest that Wnt signaling might be down-regulated during aging, leading to increased vulnerability of the neural network and increasing the risk for the onset and progression of age-related pathologies, such as AD. Considering that Wnt signaling activation attenuates the cognitive decline observed in the rodent adult brain (Toledo and Inestrosa, 2010; Vargas et al., 2014), it is likely that the modulation of endogenous Wnt signaling components might represent a promising strategy to achieve healthy aging (Gammons and Bienz, 2018; Palomer et al., 2019).

Interestingly, during the last decade several studies have identified the *Octodon degus*, a South American rodent endemic to Central Chile, as a model that naturally develops several molecular and physiological hallmarks attributable to neuropathological changes, including neuronal plasticity decrease, cognitive decline, and neuroinflammation (Inestrosa et al., 2005; Rivera et al., 2016; Cisternas et al., 2018; Lindsay et al., 2020). Remarkably, these events resemble the molecular features observed during AD development, suggesting that *O. degus* may constitute a more reliable model of this pathology (Inestrosa et al., 2005; Cisternas et al., 2018).

Thus, in the present work we studied the brain expression and activity of several Wnt signaling components, critical for the proper functioning of this pathway, during the aging of *O. degus.* We observed in both, cortex and hippocampus, a significant decrease in the expression of several Wnt ligands and Wnt components in an age-dependent manner. These results were correlated with a decrease in the expression of Wnt target genes. Together, our results are consistent with the idea that the loss of function of the Wnt signaling pathway is a feature of the aged brain and it might be responsible, at least in part, for the cognitive deficits observed in aged rodents (Oliva et al., 2018).

## Materials and Methods

### Animals

*Octodon degus* were obtained from a breeding colony at the animal facility of the Universidad de Valparaiso, Chile, and were maintained in a controlled temperature room (23 ± 1°C) under a 12:12 light/dark cycle with water and food *ad libitum. O. degus* of either sex were grouped by age: 7 to 72 months old, where no differences were observed between males and females animals. *O. degus* live on average 7 years in captivity, making it a useful model for longitudinal studies (Lee, 2004). As well as in our study, former researchers in the laboratory have classified the *O. degus* age-groups in young (1–2 years), adult (3–5 years old), and old (6 years old or more; Inestrosa et al., 2015). This classification was made based on previous studies performed in *O. degus*. van Groen et al. (2011) classified them in young (1 year old), adult (3 years old), and aged (6 years old; van Groen et al., 2011), and Du et al. (2015) divide them in young (average 1 year old), adult (average 2 years), and old (average 6 years; Du et al., 2015).

Another group of adult female *O. degus* (56 months old) and young female *O. degus* (12 months old) obtained from our colony at Faculty of Biological Sciences, Pontificia Universidad Católica de Chile were also used. These animals were all derived from laboratory-bred lines. *O. degus* were randomly divided into three groups (*n* = 8 per group) with bedding of hardwood chips and with water and food *ad libitum*. For the appropriated group, intraperitoneal (IP) injections were administered as previously described by our laboratory. Briefly, 2.0 or 4.0 mg/kg Andrographolide (ANDRO) from Sigma Aldrich was injected in saline vehicle, administered 3 times per week during 3 months. Control animals were injected with only vehicle (saline solution). Each week, we measured body mass, and the doses for IP injections were recalculated. All experiments followed the guidelines of the National Institutes of Health (NIH, Baltimore, MD, United States). All procedures were approved by the Bioethical and Biosafety Committee of the Faculty of Biological Sciences of the Pontificia Universidad Católica de Chile (CBB-121-2013). All efforts were made to minimize animal suffering and to reduce the number of animals used.

### Perfusion

All animals (young and aged) were anesthetized with Equitesin (2.5 ml/kg, i.p.) and injected with heparin (4 USP/kg, i.p.) before perfusion. Afterward, they were perfused through the heart with perfusion buffer containing 0.1% sodium nitrite, followed by fixation with 4% *p*-formaldehyde in 0.l M phosphate buffer (PB) for 30 min. Brains were surgically removed and post-fixed in the same fixative for 3 h at room temperature, followed by storage in 10% sucrose in phosphate-buffered saline (PBS) at 4°C overnight. After fixation, brains were cooled to ensure unbiased processing and analysis. The brains were subdivided into three coronal parts (approximately 3 mm in size): frontal, medial, and caudal areas. Each area was sectioned into 36 coronal sections 50 μm thick with a cryostat at −20°C.

### Immunofluorescence

Immunofluorescence (IF) of brain sections was performed as described previously (Lindsay et al., 2020). After PBS and PBS-T washes, brain sections were incubated in 0.15 M glycine, and 10 mg/ml NaBH_4_ to diminish background auto-fluorescence. Sections were washed with PBS and PBS-T and blocked with 3% bovine serum albumin (BSA) at room temperature for 1.5 h to avoid non-specific binding. Detection of the target protein was performed using a corresponding primary antibody, incubated overnight at 4°C in PBS-T containing 0.5% BSA. After washing with PBS-T, sections were incubated for 2 h at room temperature with a secondary antibody in PBS-T containing 3% BSA. Then, they were washed with PBS-T, PBS, and water and mounted on gelatin-coated slides. Coverslips with fluorescence mounting medium were added. The following primary antibodies were used: rabbit anti-phospho-S9 GSK-3β (9336) from Cell Signaling, United States (1:50), mouse anti-phospho-Y216-GSK-3β (13A) from BD Bioscience, United States (1:50), rabbit anti-Dkk-1 (sc-25516) from Santa Cruz Biotechnology, United States (1:200), rabbit anti-SFRP-1 (ab4193) from Abcam, United Kingdom (1:200), and rabbit anti-SFRP-2 (ab111874) from Abcam, United Kingdom (1:200).

### Westernblotting

The brains of animals were dissected on ice and were processed or frozen at –150°C. Briefly, hippocampal tissue was homogenized in RIPA buffer (50 mM, Tris–Cl, pH 7.5, 150 mM NaCl, 1% NP-40, 0.5% sodium deoxycholate, and 1% SDS) supplemented with a protease inhibitor cocktail (Sigma-Aldrich P8340) and phosphatase inhibitors (50 mM NaF, 1 mM Na_3_VO_4_, and 30 μM Na_4_P_2_0_7_) using a Potter homogenizer. The homogenate was then passed through different caliber syringes. Protein samples were centrifuged at 14000 rpm at 4°C twice for 15 min (Tapia-Rojas et al., 2016; Tapia-Rojas and Inestrosa, 2018). Protein concentration was determined using a BCA Protein Assay Kit (Pierce Biotechnology, Rockford, IL, United States). A total of 20 μg of whole hippocampal or cortex samples was resolved by 10% SDS-PAGE and transferred to a PVDF membrane. The reactions were followed by incubation with a primary antibody, incubation with a secondary peroxidase-conjugated antibody (Pierce), and development of the membranes using an enhanced chemiluminescence (ECL) kit (Western Lightning Plus ECL, PerkinElmer). The rabbit anti-Wnt3a (ab28472; 1:1000), rabbit anti-phospho-Ser235 *tau* (ab30664; 1:1000), rabbit anti- phospho-Thr-231 *tau* (ab30665), rabbit anti-SFRP-1 (ab4193; 1:500), rabbit anti-SFRP-2 (ab111874; 1:500), and rabbit anti-CAMKIV (ab3557; 1:1000) primary antibodies were purchased from Abcam, United Kingdom. Goat anti-Wnt7a (sc-26361), mouse anti-Dvl3 (sc-8027; 1:200), mouse anti-GSK-3β (sc-9166; 1:1000), rabbit anti-Dkk-1 (sc-25516), rabbit anti-c-jun (sc-1694), mouse anti-CyclinD1 (sc-450; 1:1000), mouse anti-TAU (sc-5587), and mouse anti-β-catenin (sc-7963; 1:500) were purchased from Santa Cruz Biotechnology, United States. Rabbit anti-phospho-S9 GSK-3β (9336; 1:1000), and rabbit anti-phospho-Ser33/37/Thr41β-catenin (9561) were purchased from Cell Signaling, United States. Goat anti-Wnt-5a (AF645; 1:1000) was purchased from R&D Systems United States. Mouse anti-Actin (11978) was purchased from Sigma-Aldrich, United States (1:10000) and mouse anti-phospho-Y216-GSK-3β (13A) was purchased from BD Bioscience, United States (1:1000).

### Image Analysis

Stained brain sections were photographed using an Olympus BX51 microscope coupled to a Micro-publisher 3.3 RTV camera (QImaging). The luminescence of the incident light and the time of exposure were calibrated to assign pixel values ranging from 0 to 255 in RGB images (no-light to full-light transmission) and was used in all preparations. The images were loaded into ImageJ v.1.40 *g* software (NIH) for analysis. The selection of areas for measurement was performed by manual threshold adjustment or by direct manual selection of regions of interest (ROIs) in heterogeneous stains. IF images of neurons were captured with a Zeiss LSM 5 Pascal confocal microscope. We typically examined a series of 15–20 confocal layers representing fluorescence data from the region of interest.

The quantification of the images was performed using the average signal intensity per area. Additionally, statistical analyses include the normalization of the data, where the value (average signal intensity per area) obtained for each slice (from old and young animals) is then divided by the average value of the young slices. By using this method, young value reaches always the normalized value 1, and able us to perform analyses based on the fold-of-change between young and old animals.

### Preparation of Images

Digital images were obtained using Adobe Photoshop 7.0. General adjustments in color, contrast and brightness were performed, and images were converted into figures.

### Statistical Analysis

Results are expressed as the mean ± standard error of the mean. Data were analyzed by one-way ANOVA, followed by Bonferroni’s *post hoc* test. Statistical significance was set at *p* ≤ 0.05. Statistical analysis was performed using Prism software (GraphPad Software Inc).

## Results

### Wnt Ligands Decline With Age in the Brain of *O. degus*

**FIGURE 1:**
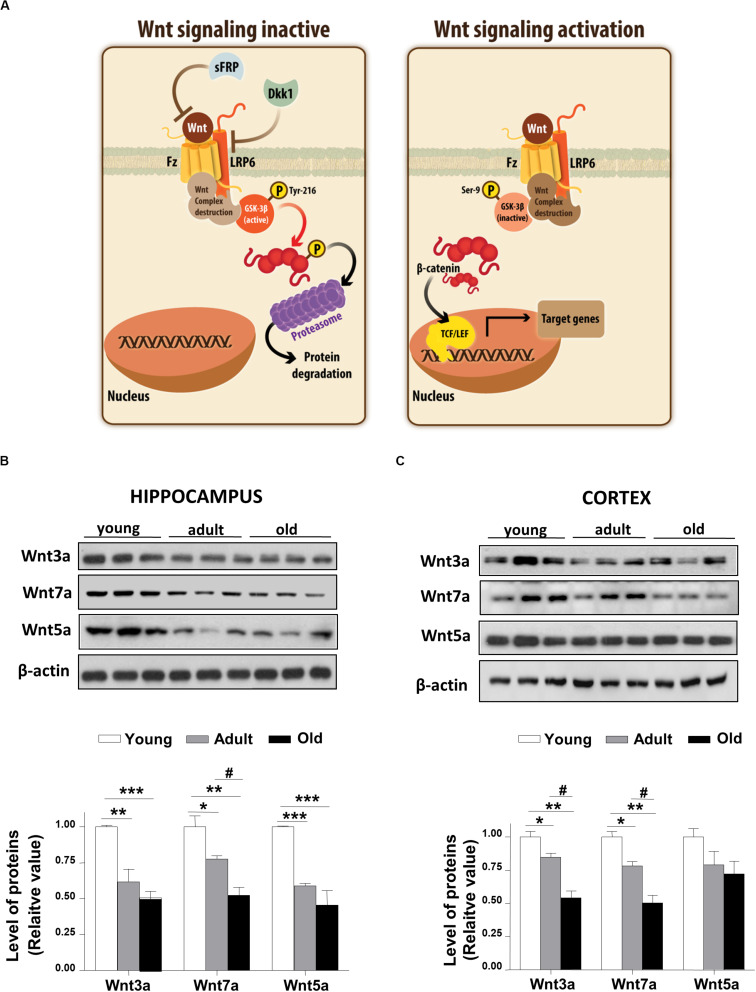
Wnt ligand levels decline in the brains of *O. degus* at different ages. **(A)** *Scheme of the Wnt signaling inactive*, we observed that GSK-3β phosphorylate β-catenin, which is eventually labeled for destruction in the proteasome. *Scheme of Wnt signaling activation*, here the Wnt ligand interacted with the Frizzled receptor and the co-receptor LRP6, which activates the intracellular signaling, GSK-3β is inhibited and the destruction complex is separated, then β-catenin translocates to the nuclei where activate the transcription of Wnt target genes. The levels of the Wnt ligands Wnt3a, Wnt7a, and Wnt5a were detected in **(B)** the hippocampus and **(C)** the cortex of *O. degus* at different ages (young: between 7 and 12 months old, adult: between 24 and 48 months old, and old: between 60 and 72 months old) by western blot analysis. Densitometric analysis of the western blots is shown below each one. Data are presented as the mean ± S.E.M. of measurements from three animals. Differences were evaluated by ANOVA, followed by Bonferroni´s *post hoc* test. Asterisks indicate significance of the observed differences (^###/***^p < 0.001; ^##/**^p < 0.01; and ^#/*^p < 0.05).

**FIGURE 2:**
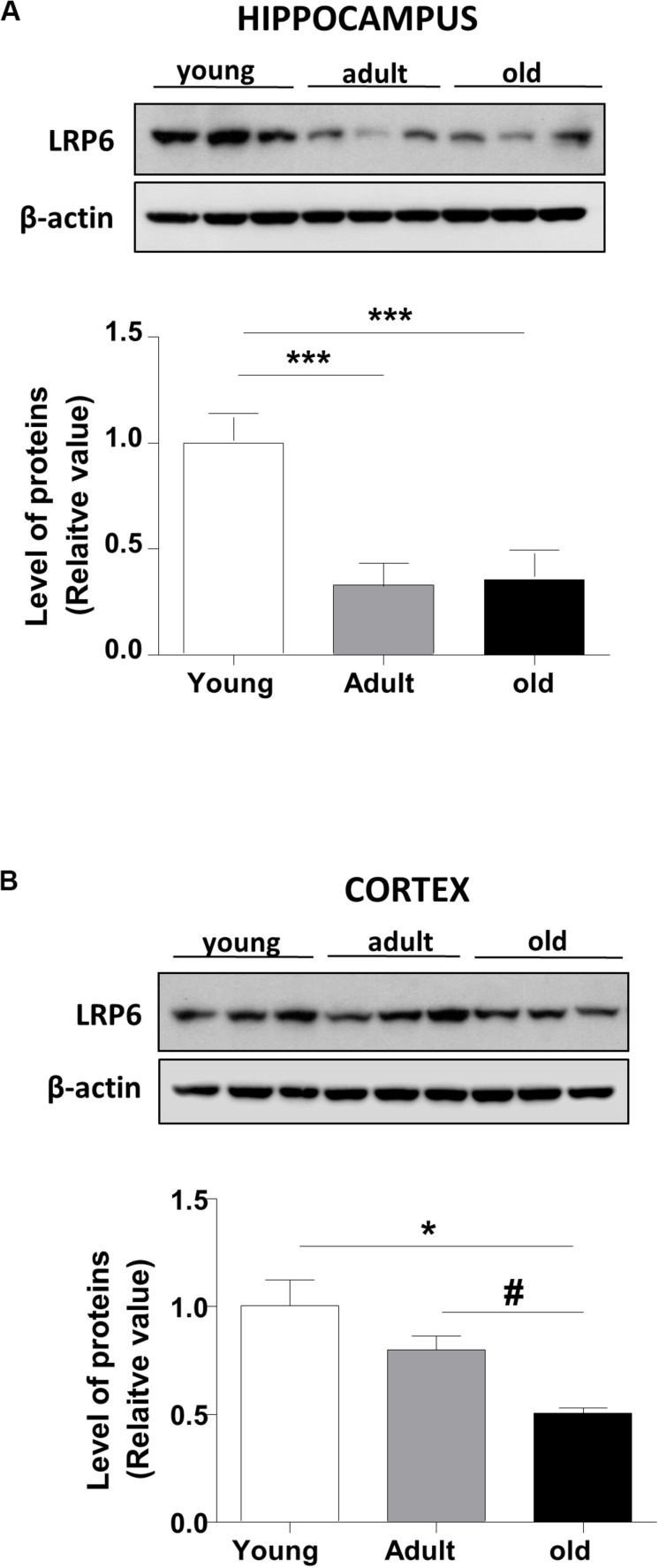
LRP6 levels changes in the brains of *O. degus* at different ages. The level of the co-receptor LRP6 as detected in **(A)** the hippocampus and **(B)** the cortex of *O. degus* at different ages (young: between 7 and 12 months old, adult: between 24 and 48 months old, and old: between 60 and 72 months old) by western blot analysis using a specific antibody. Densitometric analysis of the western blots is shown below each one. Data are presented as the mean ± S.E.M. of measurements from three animals. Differences were evaluated by ANOVA, followed by Bonferroni’s *post hoc* test. Asterisks indicate significance of the observed differences (^###/***^p < 0.001; ^##/**^p < 0.01; and ^#/*^p < 0.05).

Wnt ligand activates the canonical Wnt pathway by binding to LRP6 and Frizzled receptors, leading to the stabilization of β-catenin. In turn, the stabilized β-catenin translocates to the nucleus where it binds to the TCF/LEF transcription factor inducing the expression of Wnt target genes (Nusse and Varmus, 2012; Figure 1A). To address whether Wnt signaling is deregulated during aging in the brain of *O. degus*, through immunoblotting we evaluated the protein levels of the Wnt ligand in the whole cortex and the hippocampus at different ages. We studied the canonical ligands Wnt3a and Wnt7a and the non-canonical Wnt5a ligand, which are highly expressed in the brain. Our results indicate that adult *O. degus* exhibited decreased protein levels of the three ligands in the hippocampus. Indeed, old *O. degus* displayed a greater decrease in the expression of the ligands compared to adult animals (Figure 1B). However, at the cortex, although a significant decrease in the levels of Wnt3a and Wnt7a ligands in adult and old *O. degus* was observed, the Wnt5a expression remaining unchanged (Figure 1C). Taken together, these results indicate that Wnt ligands protein levels decrease in the brain *O. degus* during aging. Similarly, when we evaluated the protein abundance of the Wnt ligand co-receptor, LRP6, in young, adult and old *O. degus*, a significant decrease was observed in the hippocampus of the adult and old animals (Figure 2A). In the cortex, however, the levels of LRP6 diminished only in old *O. degus* (Figure 2B).

### GSK-3β Activity Increased in the Brain of Aged *O. degus*

**FIGURE 3:**
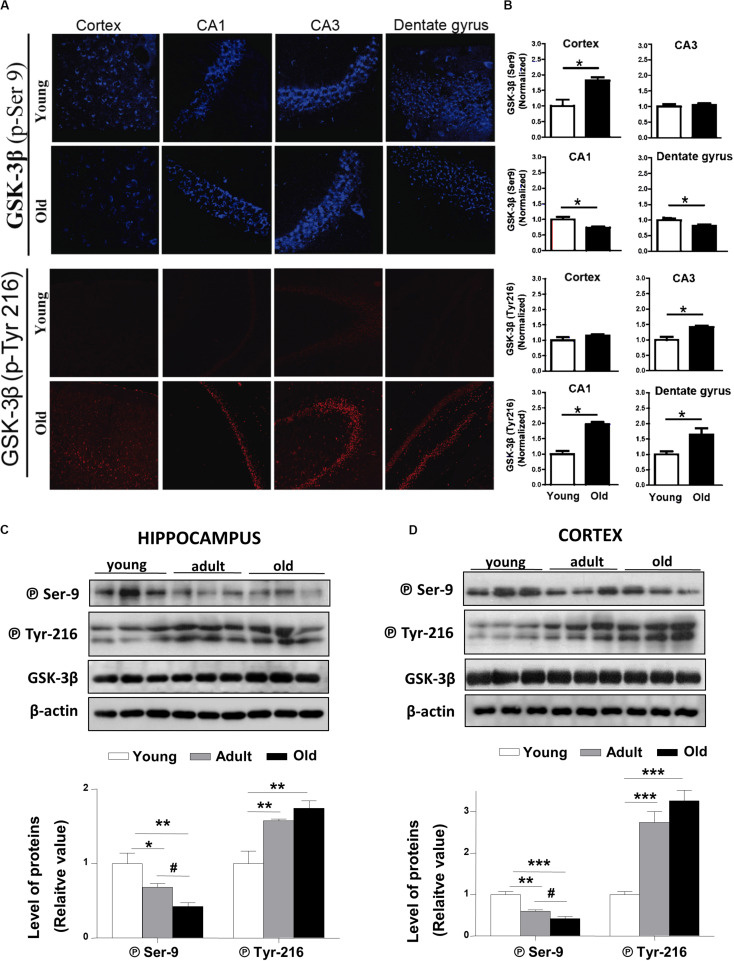
GSK-3β phosphorylation states are altered in the brains of *O. degus* at different ages. **(A)** Representative cytochemical micrographs of GSK-3β phosphorylated at Ser9 (blue, *upper panel*) and Tyr216 (red, *lower panel*) in brain slices of *O. degus*; the cortex and hippocampus (CA1, CA3, and dentate gyrus regions) are shown. **(B)** Quantification of the images in **(A)**. Western blot analysis using antibodies directed against phosphorylated GSK-3β (phospho-Ser9 and –Tyr216) and total GSK-3β in **(C)** the hippocampus and **(D)** the cortex of *O. degus* at different ages (young: between 7 and 12 months old, adult: between 24 and 48 months old, and old: between 60 and 72 months old). Quantification of the western blots is shown below. Data are presented as the mean ± S.E.M. of measurements from three animals. Differences were evaluated by ANOVA, followed by Bonferroni´s *post hoc* test. Asterisks indicate significance of the observed differences (^###/***^p < 0.001; ^##/**^p < 0.01; and ^#/*^p < 0.05).

The activation of the canonical Wnt signaling triggers downstream the inactivation of the Glycogen Synthase Kinase-3β (GSK-3β; Nusse and Varmus, 2012). In the *O. degus* brain, two phosphorylated forms of GSK-3β are present. Phosphorylation of GSK-3β at serine 9 (P⃝ Ser9) leads to the inactive form of the enzyme, while GSK-3β phosphorylated at tyrosine 216 (P⃝-Tyr216) corresponds to the active form of the enzyme (Giese, 2009). Our IF results show that the levels of the inactive form of GSK-3β (P⃝ Ser9), slightly decreased in the dentate gyrus and the CA1 hippocampal region, and did not change in the cortex and CA3 regions of the hippocampus with advanced aging (Figures 3A,B, *upper panels*). By contrast, the levels of active GSK-3β (P⃝ Tyr 216) were clearly increased in all the hippocampal regions studied (Figure 3A, *lower panels*). Additionally, we measured the protein levels of inactive GSK-3β (P⃝ Ser9) using western blotting in total cortical and hippocampal extracts (Figures 3C,D). The levels of the inactive form of GSK-3β gradually decreased in adult and old *O. degus* in both brain areas. Conversely, the expression of the active form of GSK-3β (P⃝ Tyr216) increased similarly in adult and old animals compared to younger animals in both cortex and hippocampus (Figures 3C,D). These results indicate that during aging *O. degus* increase the activity of GSK3β in both hippocampus and cortex.

### Wnt Signaling Effector Changes in the Aging Brain of *O. degus*

**FIGURE 4:**
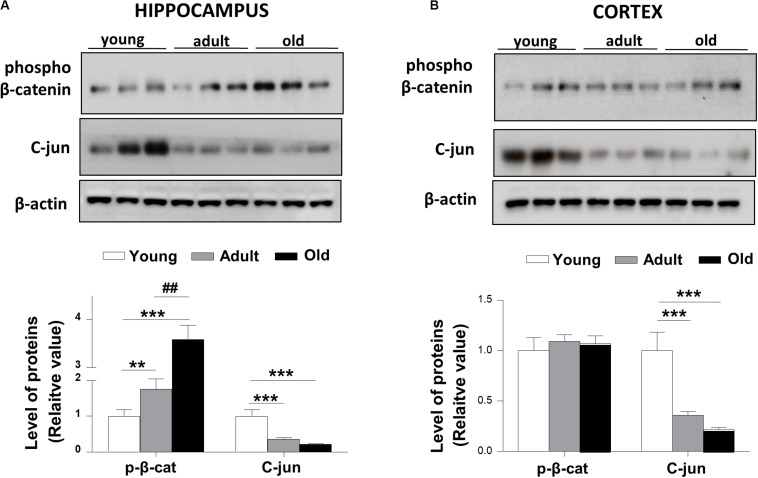
Phospho-β-catenin levels are increased and c-Jun target gene protein levels decreased in the brains of *O. degus* at different ages. Western blot analysis in **(A)** the hippocampus and **(B)** the cortex of *O. degus* (young, adult and old) using antibodies directed against phosphorylated β-catenin and c-jun (a Wnt target gene). Densitometric analysis of the western blots is shown below. Data are presented as the mean ± S.E.M. of measurements from three animals. Differences were evaluated by ANOVA, followed by Bonferroni´s *post hoc* test. Asterisks indicate significance of the observed differences (^###/***^p < 0.001; ^##/**^p < 0.01; and ^#/*^p < 0.05).

We measured the levels of β-catenin phosphorylated at the Ser33/Ser37/Thr41 sites, which are associated with GSK-3β regulation to promote the proteasome degradation of β-catenin. Consistent with previous works (Ghanevati and Miller, 2005) we found that phospho-β-catenin protein levels were increased in old *O. degus* compared with young and adult animals in the hippocampus (Figure 4A). However, we did not observe a significant change in the levels of phospho-β-catenin in the cortex of adult and old *O. degus* (Figure 4B). Considering that phospho-β-catenin is degraded via the proteasome and is not available for the activation of Wnt target genes, we measured the levels of c-jun protein, a target gene of the canonical Wnt signaling (Oliva et al., 2018). We observed that c-jun levels were significantly reduced in adult and old *O. degus* in the hippocampus and the cortex (Figures 4A,B), in agreement with the decreased LRP6 in both cortex and hippocampus and increased phospho-β-catenin levels observed in the hippocampus.

### The Protein Levels of the Wnt Antagonist Dkk-1 and SFRP Increase in the Brain of Aged *O. degus*

**FIGURE 5:**
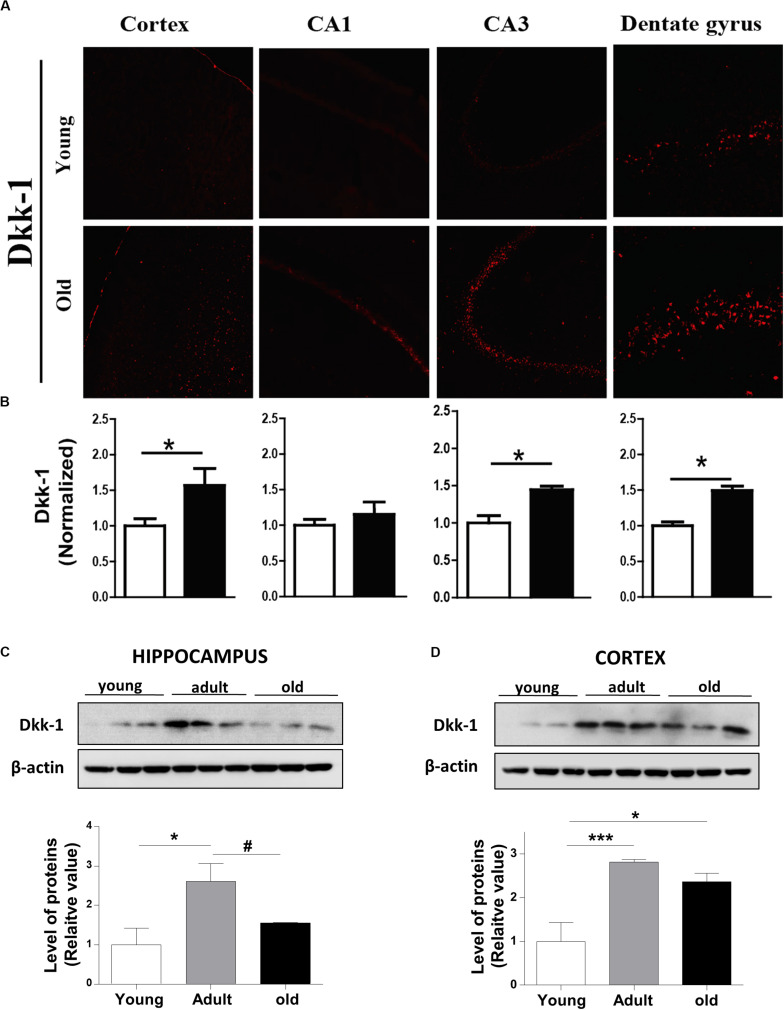
The protein levels of Dkk*-1*, a negative modulator of Wnt signaling, are increased with the age in the brains of *O. degus*. **(A)** Representative cytochemical micrographs of Dkk-1 expression (red) in brain slices of *O. degus*; the cortex and the hippocampus (CA1, CA3, and dentate gyrus regions) are shown. **(B)** Quantification of the images in **(A)**. Western blot analysis of Dkk-1 levels in **(C)** the hippocampus and **(D)** the cortex of *O. degus* at different ages (young: between 7 and 12 months old, adult: between 24 and 48 months old, and old: between 60 and 72 months old). Quantification of the western blots is shown below. Data are presented as the mean ± S.E.M. of measurements from three animals. Differences were evaluated by ANOVA, followed by Bonferroni´s *post hoc* test. Asterisks indicate significance of the observed differences (^###/***^p < 0.001; ^##/**^p < 0.01; and ^#/*^p < 0.05).

Dickkopf -1 (Dkk-1) is a secreted glycoprotein that inhibit the canonical Wnt signaling pathway by binding to the LRP6 co-receptor, thereby preventing the formation of the Wnt-Fz-LRP6 complex required for the activation of Wnt signaling (Ahn et al., 2011). In this context, we evaluated whether Dkk-1 protein levels change during aging in *O. degus*. Our results indicated that Dkk-1 is up-regulated in an age-dependent manner in both cortex and hippocampus. Through IF assays, we observed that the levels of this protein were significantly elevated in the cortex, dentate gyrus and CA3 region of old *O. degus* compared to young animals (Figures 5A,B). Western blot analysis further indicated that Dkk-1 protein was increased only in the adult hippocampus, and in both the adult and old cortex (Figures 5C,D). Increased Dkk-1 protein levels are likely to result in the inhibition of the canonical Wnt pathway (Niehrs, 2006; Purro et al., 2012). Therefore, the increase in Dkk-1 observed in the present work provides additional supporting evidence to suggest that the activity of the Wnt signaling pathway decreases in the brain of aged *O. degus.*

**FIGURE 6:**
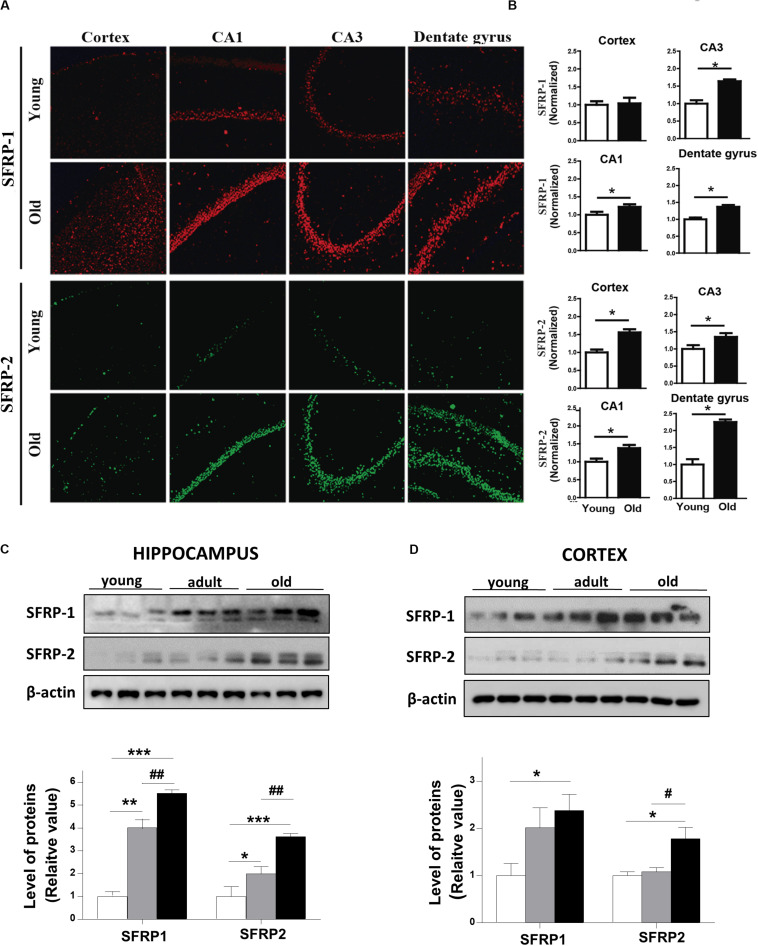
Protein levels of SFRPs, antagonists of Wnt signaling, are increased in brains of aged *O. degus*. **(A)** Representative micrographs of SFRP-1 (red, *upper panel*), and SFRP-2 (green, lower panel) protein expression in brain slices of *O. degus*; the cortex and the hippocampus (CA1, CA3, and dentate gyrus regions) are shown. **(B)** Quantification of the images in **(A)**. Western blot analysis of both SFRP-1 and SFRP-2 protein levels in **(C)** the hippocampus and **(D)** the cortex of *O. degus* at different ages (young: between 7 and 12 months old, adult: between 24 and 48 months old, and old: between 60 and 72 months old). Quantification of the western blots is shown below. Data are presented as the mean ± S.E.M. of measurements from three animals. Differences were evaluated by ANOVA, followed by Bonferroni´s *post hoc* test. Asterisks indicate significance of the observed differences (^###/***^p < 0.001; ^##/**^p < 0.01; and ^#/*^p < 0.05).

On the other hand, secreted scavenger-antagonists also regulated Wnt signaling activity by direct interaction with Wnt ligands. These inhibitors encode secreted frizzled-related proteins (SFRPs), which have an N-terminal cysteine-rich domain (CRD) with a sequence similarity with the Frizzled receptors (Bafico et al., 1999; Cruciat and Niehrs, 2013). We determined the protein levels of SFRP-1 and SFRP-2 in the brain of young and aged *O. degus* by IF. Our data show that SFRP-1 is increased in different regions of the hippocampus of aged animals compared to young animals (Figures 6A,B, *upper panels*). Also, a significant increase in SFRP-2 protein was detected in aged *O. degus* in both the cortex and the three hippocampal regions analyzed (Figures 6A,B, *lower panels*). Consistently, we found that SFRP-1 and SFRP-2 are both gradually up-regulated in the cortex and the hippocampus of *O. degus* with the age, according to western blot analysis (Figures 6C,D).

### ANDRO Recovers the Wnt Signaling Loss in Adult *O. degus* Brain

**FIGURE 7:**
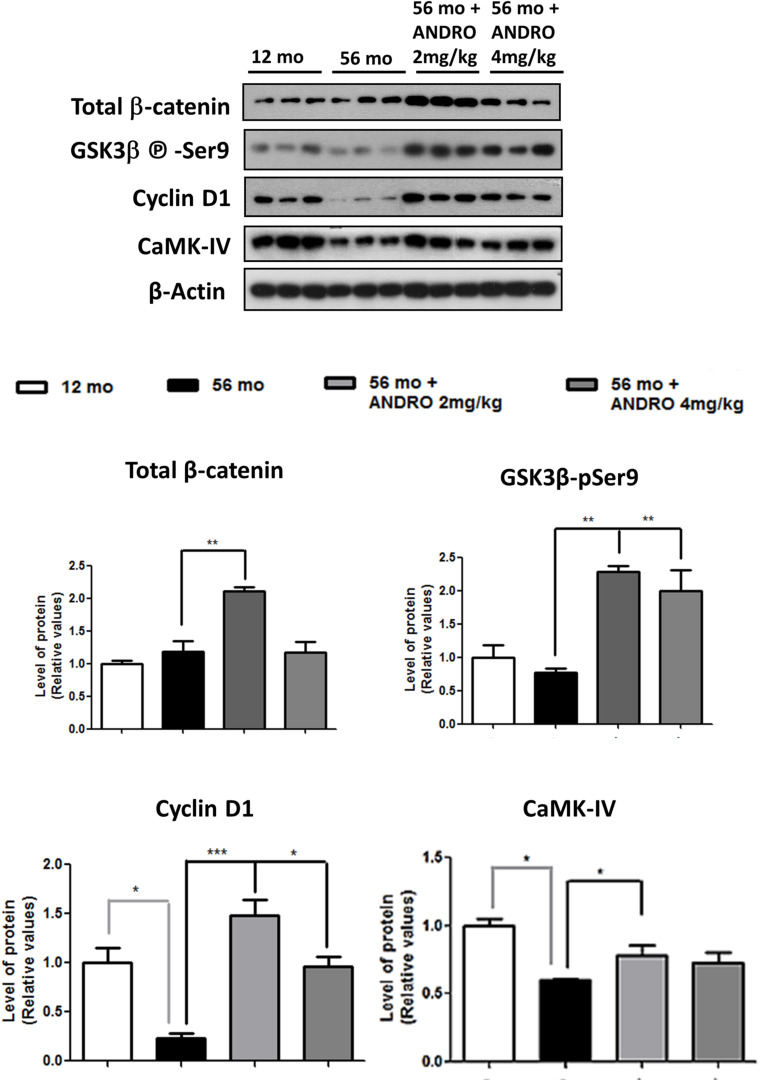
ANDRO treatment recovers the Wnt signaling loss presented in adult *O. degus*. Western blot analysis in the hippocampus of *O. degus* (young-12 months old, adult-56 months old, adult-56 months old treated with ANDRO 2 and 4 mg/kg) using antibodies directed against β-catenin, GSK3β-phospho Ser9, Cyclin D, CAMK IV, and β-actin. Densitometric analysis of the western blots is shown below. Data are presented as the mean ± S.E.M. of measurements from three animals. Differences were evaluated by ANOVA, followed by Bonferroni´s *post hoc* test. Asterisks indicate significance of the observed differences (^###/***^p < 0.001; ^##/**^p < 0.01; and ^#/*^p < 0.05).

**FIGURE 8:**
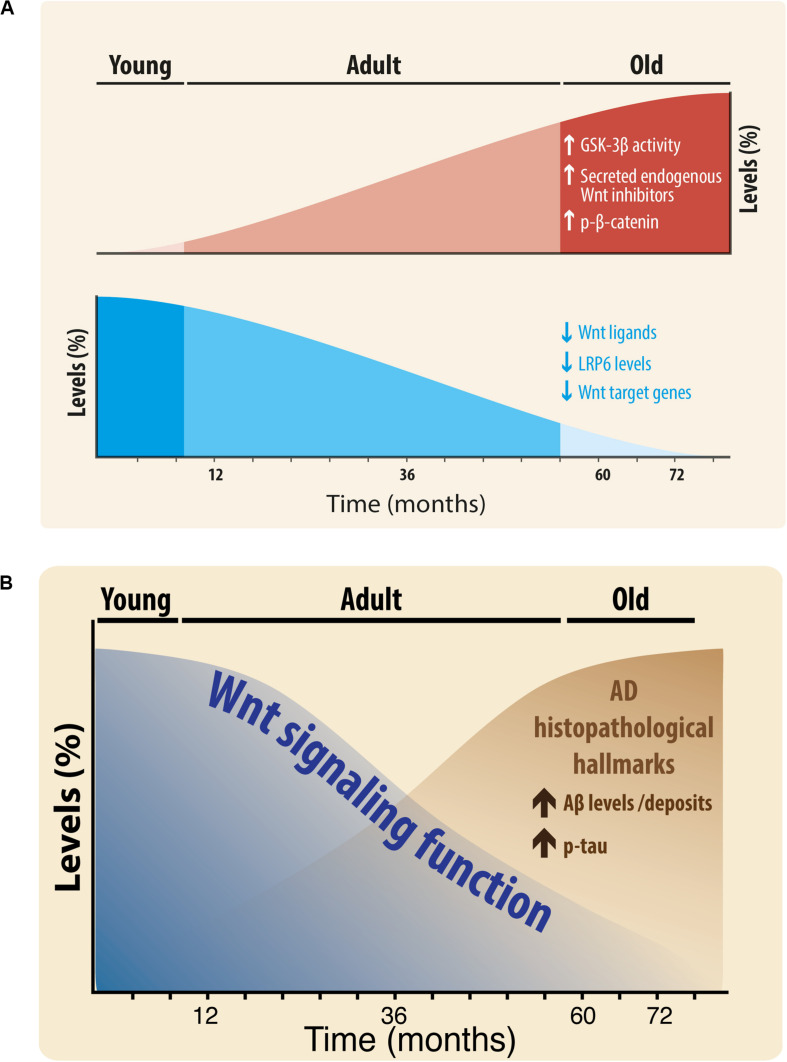
**(A)** Scheme of the Changes in Wnt signaling Components during aging of the brain of *O. degus.* Wnt components that increase during aging: GSK-3β activity, secreted endogenous Wnt antagonists, and phospho-β-catenin (red color, up). Wnt components that decrease during aging: Wnt ligands Wnt3a, Wnt7a, and Wnt5a, LRP6, and Wnt target genes (blue color, down). **(B)** Relationship of Wnt signaling function with Alzheimer’s histopathological hallmarks. Wnt signaling function decreases during aging, at the same time that key AD lesions are increased.

At present, our results described an age-dependent downregulation of components and function of Wnt signaling, mainly in the canonical pathway, of *O. degus*. In this context, we decided to study whether an activator of the Wnt canonical signaling could reestablish the protein levels and activity of Wnt components in adult animals, where the first alterations begin. In this regard, ANDRO, a bioactive molecule extracted from a medicinal plant used as pain-killer in China called *Andrographis paniculata*, is able to cross the blood brain barrier and therefore has been highly studied previously in our laboratory (Lu et al., 2019). Our results indicate that ANDRO activates Wnt signaling pathway through direct inactivation of the enzyme GSK3β (Tapia-Rojas et al., 2015). Thus, we treated the adult *O. degus* with IP injections of ANDRO and we observed recovery of various canonical Wnt signaling components. First, the β-catenin levels, a key Wnt signaling component that decreased in the aged brain, were clearly recovered after ANDRO application (Figure 7). Moreover, a significant decrease in GSK-3β activity, expressed as an increase in the Ser9 phosphorylation levels, was also observed in adult animals treated with ANDRO compared to the non-treated aged animals. Previously, we observed that a Wnt target gene, c-jun was significantly reduced with the age *O. degus* in the hippocampus and the cortex (Figure 4). Here we measured the protein levels of other two Wnt target genes, Cyclin D1 and CAMK-IV. Both proteins are decreased in the adult brain, however, after ANDRO treatment a clear recovery in Cyclin D1 and CAMK-IV protein levels were observed in *O. degus* compared with the non-treated *O. degus* (Figure 8). Altogether, these results indicate that ANDRO treatment reestablishes the protein levels of keys component of canonical Wnt signaling, strongly suggesting that the recovery of the activity of the Wnt pathway could be involved in memory improvement previously observed in O. degus treated with ANDRO (Rivera et al., 2016).

## Discussion

Wnt signaling is recognized as fundamental for both the development and function of the central nervous system (Inestrosa and Varela-Nallar, 2015). It has been demonstrated that the Wnt signaling pathway is involved in several processes necessary for the maintenance and performance of the neuronal network, including adult hippocampal neurogenesis, the establishment of the synapses, neuronal firing activity, neuronal plasticity, nerve transmission, and mitochondrial dynamics (Varela-Nallar et al., 2016; Oliva et al., 2018; Steinhart and Angers, 2018). Moreover, different canonical Wnt ligands have been demonstrated to have a direct effect on the architecture and function of the presynaptic region. The present study identified specific age-related changes in the canonical Wnt ligands (Wnt3a and Wnt7a) in both hippocampus and cortex and, to a lesser extent, in the non-canonical Wnt5a ligand (only in the hippocampus; Cerpa et al., 2008; Farias et al., 2009; Oliva et al., 2018). Wnt7a, for example, increases the formation of clusters of synaptophysin and acetylcholine receptors in hippocampal neurons (Farias et al., 2007). Wnt3a stimulates the exocytosis and recycling of synaptic vesicles in hippocampal neurons (Cerpa et al., 2008), and Wnt5a, a non-canonical ligand stimulates the postsynaptic region and PSD-95 (Farias et al., 2009; Ramos-Fernandez et al., 2019). Our results indicate an overall decrease of Wnt ligands, suggesting that Wnt-dependent synaptic stability declines with age.

Wnt ligands bind to Frizzled receptor leading to the LRP6 co-receptor recruitment, triggering the intracellular cascade that mediates synaptic stability (Liu C.C. et al., 2014). Our results also indicate a decrease in LRP6 protein levels. Considering that recent studies propose a role for LRP6 in Wnt signaling, particularly in dendritic synapse structure and long-term potentiation (LTP) in AD (Liu C.C. et al., 2014), is possible to suggest that a dysregulation of the more upstream Wnt signaling components might be related with both aging and age-related pathological conditions of the CNS in *O. degus*. Indeed, early work showed a close link between late-onset AD and the disruption of the Wnt signaling pathway in human AD patients by polymorphisms in the LRP6 gene (De Ferrari et al., 2007).

Similarly, we also observed an increase in GSK-3β activity in the brains of *O. degus* with advanced age. It is important to mention that GSK-3β activation of the rat hippocampus inhibits LTP, leading to significant synaptic impairments reminiscent of age-related neuropathology (Kremer et al., 2011). Moreover, the age-related changes observed in GSK-3β activity in *O. degus* are similar to those described during neurodegeneration in AD models (Giese, 2009; Kremer et al., 2011). Interestingly, ANDRO treatments were able to restore the Wnt signaling loss observed in the adult *O. degus* by the inhibition of GSK-3β, leading to the accumulation of β-catenin and increased expression of Wnt target genes. In regards to ANDRO treatments, we compared young and adult animals because we aimed to observe changes in the early stages of the appearance of Wnt signaling downregulation, which were observed primarily in the adult brains. In addition, we previously demonstrated that ANDRO promotes behavioral changes in the ANDRO-treated group compared to control, enhancing recognition and long-term working memory, along with improved learning performance in adult *O. degus* (Barnes Maze and NOR experiments; Rivera et al., 2016). However, the significance of our analysis relies on how Wnt signaling is affected along with the aging of this longitudinal animal model that has been proposed as an important model of AD-like neurodegeneration.

On the other hand, previous studies have also indicated that alterations in the levels of the soluble Wnt inhibitors might modulate the Wnt signaling activation (Ehrlund et al., 2013). In this regard, we observed an age-related increase in the protein levels of the Wnt antagonist SFRP-1 and SFRP-2, further suggesting that the aging-related decrease of Wnt activity is caused not only by structural component reduction or lack of activation signals, but also due to the increase of inhibitory signals. In this regard, an increase in the levels of SFRP1 has been related to cellular senescence on other cell types, including in Human Cardiac Stem Cells (Nakamura et al., 2017), and fibroblasts (Elzi et al., 2012). Recent studies also demonstrate that SFRP-1 is increased in the brain of patients with AD, binds to amyloid-β and accumulates in amyloid plaques. SFRP-1 overexpression in an Alzheimer-like mouse model anticipates the appearance of senile plaque and dystrophic neurites, whereas its genetic inactivation or the infusion of α-SFRP-1-neutralizing antibodies favors non-amyloidogenic amyloid precursor protein (APP) processing. Decreased SFRP-1 function lowers senile plaque accumulation, preventing LTP loss and cognitive deficits (Esteve et al., 2019). This study of Esteve and coworkers unveils SFRP-1 as a crucial player in AD pathogenesis through the inhibition of ADAM10, but other studies indicate that SFRP act as Wnt antagonist sequestering Wnt ligands in the extracellular space (Folke et al., 2018), therefore, these effects also could be due, almost in part, to the inhibition of Wnt signaling. More studies are necessary to validate this possibility.

Concomitantly, early evidence indicates that Dkk-1 is barely present in the healthy brain, but its protein levels are increased under pathological conditions, such as in AD (Caricasole et al., 2004). Evidence suggests that Dkk-1 is required for amyloid-β -mediated synapse loss in hippocampal neurons (Scali et al., 2006; Purro et al., 2012; Palomer et al., 2019), and its expression induces *tau* phosphorylation. Moreover, local infusion of Dkk-1 in rats caused neuronal cell death and astrogliosis in the CA1 region of the hippocampus and the death of cholinergic neurons in the nucleus basalis (Scali et al., 2006). Increased expression of Dkk-1 is causally related to neurodegeneration processes in several central nervous system disorders other than AD, such as brain ischemia and temporal lobe epilepsy (Seib et al., 2013). Moreover, dysfunctional Wnt signaling caused by increased levels of Dkk-1 has been implicated also in the age-related decline in hippocampal neurogenesis (Seib et al., 2013). As a result, mice deficient in Dkk-1 exhibit enhanced spatial working memory and memory consolidation and also show improvements in affective behavior (Caricasole et al., 2003, 2004). Accordingly, our Westernblotting (WB) data indicate that Dkk-1 is increased in the hippocampus and cortex of adult *O. degus*, possibly inhibiting Wnt signaling, this fact is consistent with previously published results obtained in different systems. However, comparing young with old animals, the differences in the cortical expression of Dkk-1 persist over time, a result contrary to our observation by IF assay. We could explain these distinct results between IF and WB based on technical differences; IF experiments allow us to sub-divide the hippocampal analyses in CA1, CA3, and DG, whereas WB were performed using the complete hippocampal tissue.

Our work shows that in agreement with previous studies, the Wnt signaling pathway is active in young *O. degus* and becomes attenuated in aged rodents. We found that the levels of certain Wnt components increase, i.e., GSK-3β activity, Dkk-1, SFRP-1, SFRP-2, and phospho-β-catenin, however, other Wnt components decrease during aging: Wnt ligands (Wnt3a, Wnt7a, and Wnt5a), LRP6, and Wnt target genes (Figure 8A). Also, is important to highlight that although the first alterations were observed in the adulthood, significant differences were also observed between adult and old brains in hippocampal Wnt7a, GSK3β-Ser9, phospho-β-catenin, Dkk-1, SFRP1, and SFRP2 proteins, in addition to significant changes in Wnt7a, Wnt3a, GSK3β-Ser9, LRP6, and SFRP2 in the cortex. Furthermore, we interpret the difference between hippocampus and cortex as a temporal-dependent difference. Previous studies indicated that AD hallmarks occur first in the hippocampus and then spread to cortex (Braak and Braak, 1997). Therefore, adult brains might show higher levels of AD-like hallmarks in the hippocampus, whereas old brains should show them in the hippocampus and cortex as we observe in our study.

Moreover, recent studies from our laboratory indicate that the inhibition of the canonical Wnt signaling induces an increase in the amyloidogenic processing of the APP, leading to an increased Aβ secretion and formation of Aβ oligomers (Tapia-Rojas et al., 2016), a critical hallmark in AD. Similarly, the Wnt signaling loss accelerates the appearance of the neuropathological hallmarks of AD in the J20-APP transgenic and wild-type mice (Tapia-Rojas and Inestrosa, 2018). Also, our results are consistent with Bai et al. (2020) in which characterizing AD stage-associated protein networks, by multi-omics, they corroborate that the Wnt pathway is associated with AD (Bai et al., 2020).

Furthermore, the treatment of aged *O. degus* with ANDRO has shown protection from several aspects of AD-pathogenesis: i.e., decreased Aβ accumulation and lower *tau* phosphorylation, recovery of synaptic protein loss and cognitive impairment (Serrano et al., 2014; Rivera et al., 2016). Moreover, several studies using ANDRO treatments had reported other effects such as adult neurogenesis (Varela-Nallar et al., 2016), neurite out-growth (Xu et al., 2019), and neuroprotection (Lindsay et al., 2020), along with decreased neuroinflammation, oxidative stress, and synaptic dysfunction in aged animals (Serrano et al., 2014; Lu et al., 2019; Zolezzi and Inestrosa, 2019; Lindsay et al., 2020). Specifically, ANDRO activates Wnt signaling pathway by inhibiting directly GSK-3β (Tapia-Rojas et al., 2015), but also it has been described as a modulator of other cellular signaling including the BACE1-dependent amyloid processing, the Nrf2-mediated p62, the Keap1/Nrf2/ARE/HO-1, the PI3K-Akt, and the NF-kB pathways (Seo et al., 2017; Gu et al., 2018, 2019; Panche et al., 2019). All these signaling pathways, complimentarily to the Wnt signaling, could be increasing the beneficial effects related to Wnt signaling modulation (Zolezzi and Inestrosa, 2019), and should be assessed in the future to better understand the mechanisms underlying ANDRO’s effects.

Taken together, our results suggest that during the aging process the Wnt signaling function decreases in the brain of the *O. degus*. Moreover, considering our previous work, we suggest that this decrease is inverse to what observed under neuropathological conditions, such as in AD, where the expression of key AD lesions increases (Figure 8B). Additionally, considering that ANDRO, is able to rescue Wnt signaling impairment, at the levels of β-catenin, GSK-3β and target genes, we can hypothesize that Wnt signaling might play a pivotal role not only in the aging process itself, but influencing the outcome of such process in terms of an improved healthy aging.

## Data Availability Statement

The raw data supporting the conclusions of this article will be made available by the authors, without undue reservation.

## Ethics Statement

The animal study was reviewed and approved by Bioethical and Biosafety Committee of the Faculty of Biological Sciences of the Pontificia Universidad Católica de Chile (CBB-121-2013).

## Author Contributions

NI conceived the research, projected the experimental approach, and wrote the manuscript. CT-R and CL conducted the experiments and process the data. NI and JZ discussed and elaborated the final version of the manuscript. All authors contributed to the article and approved the submitted version.

## Conflict of Interest

The authors declare that the research was conducted in the absence of any commercial or financial relationships that could be construed as a potential conflict of interest.
